# Editorial Department

**Published:** 1853-04

**Authors:** William H. Dwinelle


New York, April 7th, 1853.

Dr. Harris :

Dear Sir—As a wrong impression seems to have been made in the minds of some, by my statement in the Journal of January last, of a conversation had with Dr. Ehrick Parmly while I was in Paris last season, in relation to his connection with the Syracuse College, and as I have recently obtained information which will enable me to place the matter in its true light, in justice to all parties concerned, I desire to do so. The statement in the Journal is as follows : "Dr. P. intends prosecuting his studies in Paris for about a year longer. Since my return, I see by a circular from the New York Dental College at Syracuse, that Dr. Ehrick Parmly is numbered among the faculty of that Institution for the present winter course. This is probably a mistake, as he informed me while I was in Paris, that his interests had required him to decline the appointment."

Our conversation on the subject was of a general character, he simply stating to me in general terms and without qualification, that he had declined the appointment ; my impressions were general, and I so recorded them.

I see, however, by a letter from Dr. Ehrick Parmly, now in Paris, just placed in my hands, that he only intended to convey to me that he had declined the appointment for the "present winter course," now past.

Dr. P. also states in his letter, that the announcement of his name in connection with the faculty of the N. Y. Dental College for the past winter's course, was justifiable under the circumstances.

Please publish this letter in the next number of the Journal, and much oblige,

Yours, truly, William H. Dwinelle.

